# Microbial characteristics across different tongue coating types in a healthy population

**DOI:** 10.1080/20002297.2021.1946316

**Published:** 2021-07-26

**Authors:** Hairong Chen, Qingwei Li, Min Li, Sheng Liu, Chensi Yao, Zixiong Wang, Zhuoya Zhao, Ping Liu, Fan Yang, Xinjian Li, Jun Wang, Yixin Zeng, Xiaolin Tong

**Affiliations:** a CAS Key Laboratory of Infection and Immunity, Institute of Biophysics, Chinese Academy of Sciences, Beijing, China; b Departments of Endocrinology, Guang’anmen Hospital, China Academy of Chinese Medical Sciences, Beijing, China; c University of Chinese Academy of Sciences, Beijing, China; d CAS Key Laboratory of Pathogenic Microbiology & Immunology, Institute of Microbiology, Chinese Academy of Sciences, Beijing, China

## Abstract

**Background** The physical appearance of tongue coatings is vital for traditional Chinese medicine (TCM) to diagnose health and disease status. The microbiota of different tongue coatings could also influence coating formation and be further associated with specific diseases. Previous studies have focused on bacteria from different tongue coatings in the context of specific diseases, but the normal variations in healthy individuals remain unknown.**Aim:** We examined the tongue microbiota by metagenomics in 94 healthy individuals classified into eight different tongue types.**Results:** The overall composition of the tongue coating microbiome is not drastically different among different coating types, similar to the findings of previous studies in healthy populations. Further analysis revealed microbiota characteristics of each coating type, and many of the key bacteria are reported to be implicated in diseases. Moreover, further inclusion of diabetic patients revealed disease-specific enrichment of *Capnocytophaga*, even though the same tongue coatings were studied.**Conclusions:** This work revealed the characteristic compositions of distinctive tongue coatings in a healthy population, which serves as a basis for understanding the tongue coating formation mechanism and provides a valuable reference to further investigate disease-specific tongue coating bacterial markers.

## Introduction

While the gut microbiota has drawn tremendous attention, studies of the oral microbiota have only emerged relatively recently [1,2]. The tongue coating microbiota, an essential component of the tongue coating, represents a large microbial fraction of the body that mainly contains *Bacteroides, Fusobacteria, Actinobacteria* and *Firmicutes*. The microbiota composition is shaped by the uptake of nutrients from the host epithelium, saliva and their interactions [3]. The corresponding various colors and thicknesses of tongue coatings have long been used in traditional Chinese medicine (TCM) as indicators of the health status of an individual and to assist in the prediction or diagnosis [4] of diseases such as gastritis [5], esophagitis [6], pancreatic organ dysfunction [7] or even early-stage breast cancer [8]. Usually, according to its color, thickness and moisture appearance, tongue coating is classified into white thin (W-thin), white thin greasy (W-thin greasy), white thick greasy (W-thick greasy), white thick dry (W-thick dry), yellow thin (Y-thin), yellow thin greasy (Y-thin greasy), yellow thick greasy (Y-thick greasy), and yellow thick dry (Y-thick dry) by TCM.

There have been a number of studies investigating the microbiota characteristics with regard to specific disease types and with a strong focus on a particular coating type, taking W-thin coating in a healthy population as a control [9–12]. However, the identified marker bacteria may be disease specific and affected by different stages of disease progression, yet only a few studies have taken this into consideration [13]. The marker bacteria among distinctive tongue coating types thus could vary in different diseases. Microbiota related to halitosis were found to belong to *Alloprevotella tannerae* and *Solobacterium moorei*, tonsillitis was associated with *Corynebacterium argentoratense*, and periodontal diseases were associated with *Prevotella nigrescens* and *Capnocytophaga sputigena*. These bacteria were found to be aggregated in the yellow tongue coating (YTC) of gastritis patients [9]. Another comparison of 13 chronic erosive gastritis patients with typical YTC with 10 healthy volunteers with white tongue coating (WTC) demonstrated *Bacillus* as a potential YTC diagnostic marker [10]. A further study of 28 chronic hepatitis B (CHB) YTC patients and 25 CHB WTC patients with 22 healthy controls showed that *Proteobacteria* was enriched in YTC and correlated with higher HBV-DNA levels [11]. In 115 gastric cancer patients, *Proteobacteria* abundance declined from the W-thin group to the Y-thin group, with the lowest abundance in the Y-thick group [12]. Therefore, the representative microbes for certain tongue coatings vary among different diseases.

However, different tongue coating types also exist in the healthy population. Studying the microbial basis of tongue coating types in this population is vital in demonstrating the important roles of microbes in reflecting health status, excluding the impact of diagnosed disease. In our study, we investigated the natural distribution and microbial characteristics of different tongue coating types in a relatively large healthy population devoid of disease influences. Specifically, we divided the tongue coatings of a healthy population into eight types according to color, thickness and moisture appearance and investigated the microbiota compositions by metagenomic sequencing. The characteristics of bacteria for distinctive tongue coatings were identified, and the co-occurrence relationship between microbes was constructed. This provides a valuable reference for deciphering the tongue coating formation mechanism and facilitates future diagnosis. We further compared marker bacteria in healthy individuals and diabetic patients with the same tongue coating types, demonstrating the disease-specific effect on tongue coating microbes and highlighting the necessity of identifying marker bacteria for tongue coating types within a healthy population.

## Materials and methods

### Study participants

All participants provided written informed consent, and protocols were approved by the ethical commission of the Institute of Biophysics, Chinese Academy of Sciences. The target population was healthy residents in the Xinjiekou district, Beijing, China, with occupations including government officials, nursing home staff, hotel service staff and department store staff. Participants who reported no severe diseases relating to the heart, lung, liver, kidney, brain or other organs, with no chronic disease and no acute cardiovascular events or myocardial infarction in the past six months were included. Participants with medical histories of using glucocorticoids and antibiotics within the last three months and a long history of smoking, alcohol abuse or drug abuse were excluded. Pregnant or breast-feeding women were also excluded. All enrolled individuals had healthy oral tissues and were free of nonrestored carious lesions [14]. For Y-thick greasy tongue coating samples from diabetic patients, participants diagnosed with diabetes (criteria: typical diabetes symptoms including thirst, hunger, hyperphagia, body weight loss with a random blood glucose test result higher than 11.1 mmol/L, fasting blood sugar higher than 7 mmol/L, or 2 h oral glucose tolerance test higher than 11.1 mmol/L) with no complicated diseases were enrolled from endocrine outpatients at the Guang’anmen Hospital, China Academy of Chinese Medical Sciences. The rest of the filter criteria were as above. Tongue coating samples from four diabetic males residing in Beijing, China and aged 33, 48, 53, and 54 years were selected and subjected to further sequencing and analysis. The 20 individuals who were part of the salivary flow rate cohort were recruited from volunteers residing in Beijing at Guang’anmen Hospital, China Academy of Chinese Medical Sciences.

### Tongue imaging

**Figure 1.**
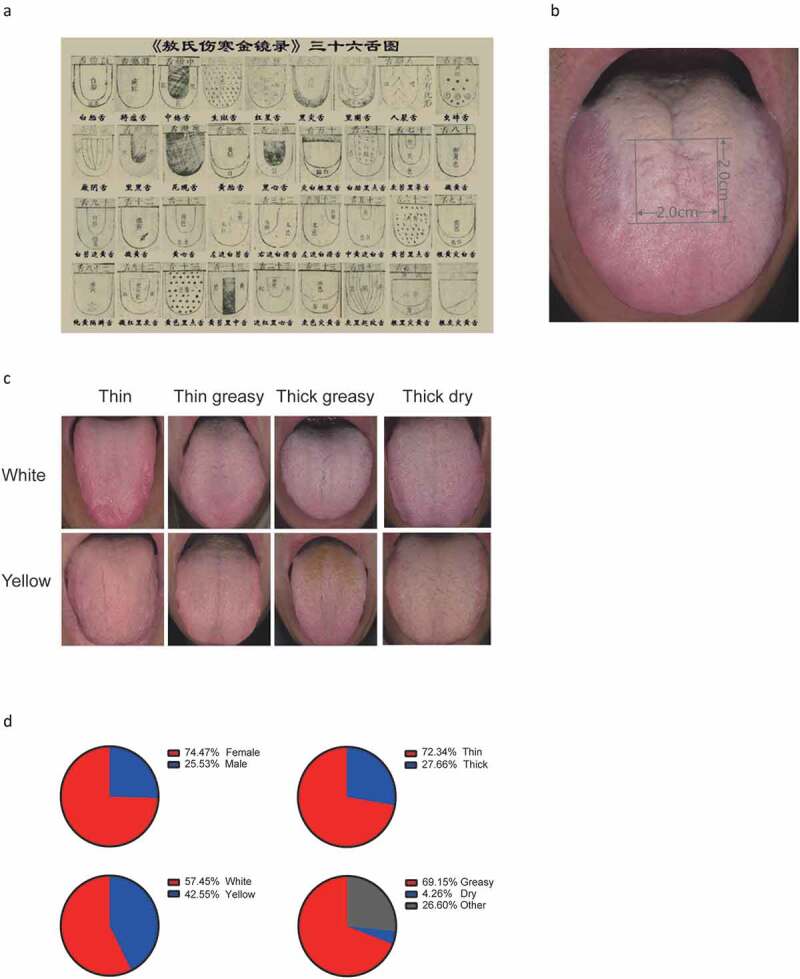
Example of traditional Chinese medicine tongue type classification and tongue-coating classification of our participants. (**A**) Thirty-six tongue types presented in the ancient medical book of Ao Shi Shang Han Jin Jing Lu (1341 A.D.). (**B**) Diagnosis and sampling site of the tongue dorsum. (**C**) Examples of eight tongue coating types (upper from left to right: white thin, white thin greasy, white thick greasy, white thick dry; lower from left to right: yellow thin, yellow thin greasy, yellow thick greasy, yellow thick dry). (**D**) Proportion of sex and specific tongue coating characteristics in the recruited healthy population

A DS01-G tongue manifestation acquisition instrument (DaoSheng Medical Technology Co. Ltd., China) was used to photograph the tongue coatings of the participants. Tongue coating types were prediagnosed at the site. The tongue coating images were then segregated into W-thin, W-thin greasy, W-thick greasy, W-thick dry, Y-thin, Y-thin greasy, Y-thick greasy and Y-thick dry by three certified and experienced TCM doctors according to the middle site of the tongue (Figure 1B). The images with consistent diagnostic results from the three TCM doctors and the doctor at the site were selected, and their corresponding samples were collected.

### Sample collection

Tongue coating samples were collected after a mouth wash and using a protocol that had been tested during a pilot phase of this study. The sampling protocol is described as follows: volunteers who met the filter criteria were required to rinse their mouths three times with mineral water at 9:00–10:00 in the morning. After resting for 30 min, tongue images were collected with a Daosheng Tongue DS01-G Image Instrument. A sterile swab was used to collect the microbiome at the middle site of the tongue. The microbiome was collected with the sterile swab by scraping back and forth 15 times, followed by a 180-degree rotation to scrape for another 15 times. Each sterile swab was stored in a TinyGene microbiome preserved Eppendorf tube containing buffer and proteinase K (TinyGen Bio-Tech (Shanghai) Co. Ltd., China) and was immediately placed on dry ice for transportation to a − 80°C freezer within two hours.

For unstimulated saliva sample collection, the participants were asked to rinse their mouths with water, followed by relaxation for 5 min, during which time a questionnaire was completed and tongue images were collected. Then, the participants were asked to swallow to void the mouth of saliva, collect the unstimulated saliva for 5 min and spit into a preweighed 15 ml Falcon tube. The salivary flow rate (g/min) was calculated as (saliva-tube weight subtracted with tube weight) g/5 min.

### DNA extraction

Total DNA was extracted from samples using protease K cleavage combined with the phenol chloroform extraction method according to the manufacturer’s protocols. DNA completeness and purity were checked by running the samples on 1.2% agarose gels. DNA concentration was checked with a Qubit fluorometer.

### Metagenomic library preparation and sequencing

Extracted DNA was sheared on a Covaris M220 machine (Covaris, USA) programmed to generate 300-bp fragments. The sequencing libraries were constructed with a NEBNext® Ultra™ DNA Library Prep Kit for Illumina® (NEB, USA). The products were purified using AgarosAgencourt AMPure XP (Beckman, USA) and quantified using the GenNext^TM^ NGS Library Quantification Kit (Toyobo, Japan). The libraries were sequenced using the Illumina NovaSeq 6000 platform and 150-bp paired-end technology at TinyGen Bio-Tech (Shanghai) Co., Ltd.

### Bioinformatics analysis

The raw FastQ files were demultiplexed based on the index. The raw, paired-end reads were trimmed and quality controlled using Trimmomatic (http://www.usadellab.org/cms/?page=trimmomatic) and cutadapt software. The host sequences were removed by using BWA (http://bio-bwa.sourceforge.net/). The clean data were assembled and predicted by the Megahit (https://github.com/voutcn/megahit) and METAProdigal (http://prodigal.ornl.gov/) software.

The predicted genes were clustered, and a nonredundant gene catalog was constructed with the CD-HIT package (http://www.bioinformatics.org/cd-hit/) (parameters: identity = 95%, coverage = 90%). The abundances of genes were estimated quickly by genomeCoverageBed in BEDTools. The representative sequences were identified taxonomically against the NR database with an e-value of 1e-5 by the DIAMOND software. The taxonomic information included domain, kingdom, phylum, class, order, family, genus, and species.

### Co-occurrence networks

Spearman’s correlation analysis and p value calculation performed with cor.test in R language were applied to the abundance values of the bacterial species with a linear discriminant analysis (LDA) value higher than 2 in each group. Bacteria with a Spearman’s correlation ≥ 0.6 and p value ≤ 0.05 were further selected for co-occurrence network construction with igraph (version 1.2.5) in R.

### Statistical analyses

Principal component analysis (PCA) was performed using *prcomp* in R with the taxonomy abundance information, and plotting was performed by ggplot2 in R (version 3.4.1). The significance of separation between each pair of groups was calculated by Analysis of Similarities (ANOSIM). The microbiota results were analyzed using the nonparametric factorial Kruskal-Wallis (KW) sum-rank test, and the LEfSe method was applied with LEfSe v1.0. p values < 0.05 were considered significant.

## Results

### Tongue coating types are unevenly distributed in the healthy population

The study population was recruited from healthy volunteers in the Xinjiekou community in Beijing. A total of 94 out of 180 individuals passed the quality filters (see Materials and methods) and were included in the tongue coating microbiome analysis. The average residence time in Beijing was approximately 20–30 years. This maximally reduced the living environmental impact on the oral microbiota, such as the composition of tap water [15]. To identify characteristic bacteria in the distinctive tongue coatings, we first classified the tongue coatings into eight types according to color, thickness and moisture appearance (Figure 1A) [16]. This resulted in W-thin, W-thin greasy, W-thick greasy, W-thick dry, Y-thin, Y-thin greasy, Y-thick greasy and Y-thick dry tongue coatings, which are the most prevalent tongue coating types. Because microbe biomass is most abundant at the middle site of the tongue dorsum, the middle site of the tongue coating (Figure 1B) was used in tongue coating typing classification (Figure 1C) and for biofilm sampling. In terms of general characteristics, the cohort contained 74.47% females and 25.53% males (Figure 1D, upper left). In total, 72.34% of the tongue coatings could be defined as thin, whereas 27.66% could be defined as a thick tongue coating (Figure 1D, upper right); the percentage of white tongue coating was highest at 57.45%, followed by that of yellow tongue coating at 42.55% (Figure 1D, lower left), while in terms of moisture, greasy tongue coating accounted for 69.15% of the population (Figure 1D, lower right).

In the recruited population, the most prevalent tongue coatings were W-thin, W-thin greasy and Y-thin greasy, numbering 20 in each, while the numbers for W-thick greasy, Y-thin and Y-thick greasy were approximately 10 cases in each. In contrast, there were a few cases of W-thick dry and Y-thick dry, with one and three cases each (Table 1). The age distribution of each group generally ranged from 45 to 55 years, with no significant differences between groups (Table 1). Of note, the one W-thick dry volunteer 36 years old and three Y-thick dry volunteers, all approximately 54 years old, were females, suggesting that rare tongue coatings are potentially age- and/or sex-related. Due to the unpaired age and few recruited numbers of W-thick dry and Y-thick dry tongue coating groups, we excluded them from the microbiota analysis. The percentage of females was highest in the W-thin group at 94.47% and lowest in the W-thick greasy group at 41.67%, suggesting different compositions of females and males in each tongue coating type (Table 1). The BMI value was highest in the W-thin greasy group at an average of 25 kg/m^2^, with no significant differences among the groups.

**Table 1.** General information on the healthy population used in our study

	White thin	White thin greasy	White thick greasy	White thick dry	Yellow thin	Yellow thin greasy	Yellow thick greasy	Yellow thick dry
Number of cases	19	22	12	1	6	21	10	3
Age	46.9 ± 6.18	44.0 ± 4.78	47.8 ± 6.18	36	46.3 ± 7.87	44.9 ± 5.53	45.7 ± 3.97	54 ± 1.73
Males(%)	1(5.26%)	5(22.73%)	7(58.33%)	0(0%)	2(33.33%)	4(19.05%)	4(40%)	0(0%)
Females(%)	18(94.74%)	17(77.27%)	5(41.67%)	1(100%)	4(66.67%)	17(80.95%)	6(60%)	3(100%)
BMI	24.0 ± 2.50	25.4 ± 3.74	24.5 ± 2.72	23.18	23.9 ± 2.38	23.9 ± 2.34	24.0 ± 2.22	23.5 ± 3.91
Years resident in Beijing	29.2 ± 18.0	29.7 ± 17.2	19.6 ± 16.2	14	29.2 ± 23.7	28 ± 17.2	20.6 ± 14.2	54 ± 1.73

Data are presented as mean ± SD

### Characteristics of the tongue coating microbiome

**Figure 2.**
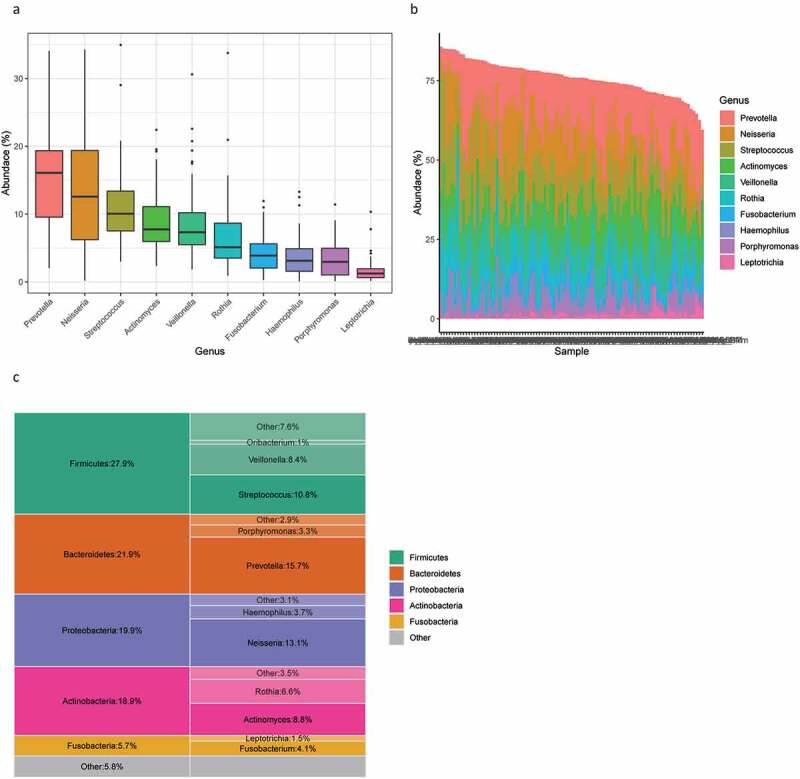
Overall microbial composition related to tongue coating. (**A**) Box plot of the ten most abundant microbes at the genus level. (**B**) Stacked bars of the ten most abundant genera in each sample. (**C**) The five most abundant phyla and their most abundant genera

Microbiome analysis revealed that the most common phylum found on the tongue was *Firmicutes*, reaching the highest abundance of 27.9%, followed by *Bacteroidetes, Proteobacteria, Actinobacteria* and *Fusobacteria*. At a finer taxonomic level, the most enriched genus of the study population was *Prevotella*, with an average abundance of 15.7%, followed by *Neisseria* at 13.1%, *Streptococcus* at 10.8%, *Actinomyces* at 8.8%, *Veillonella* at 8.4%, *Rothia* at 6.6%, *Fusobacterium* at 4.1%, *Haemophilus* at 3.7%, *Porphyromonas* at 3.3% and *Leptotrichia* at 1.5% (Figure 2A-C). The most common genus found in our study is consistent with that in other reports [11,17].

**Figure 3.**
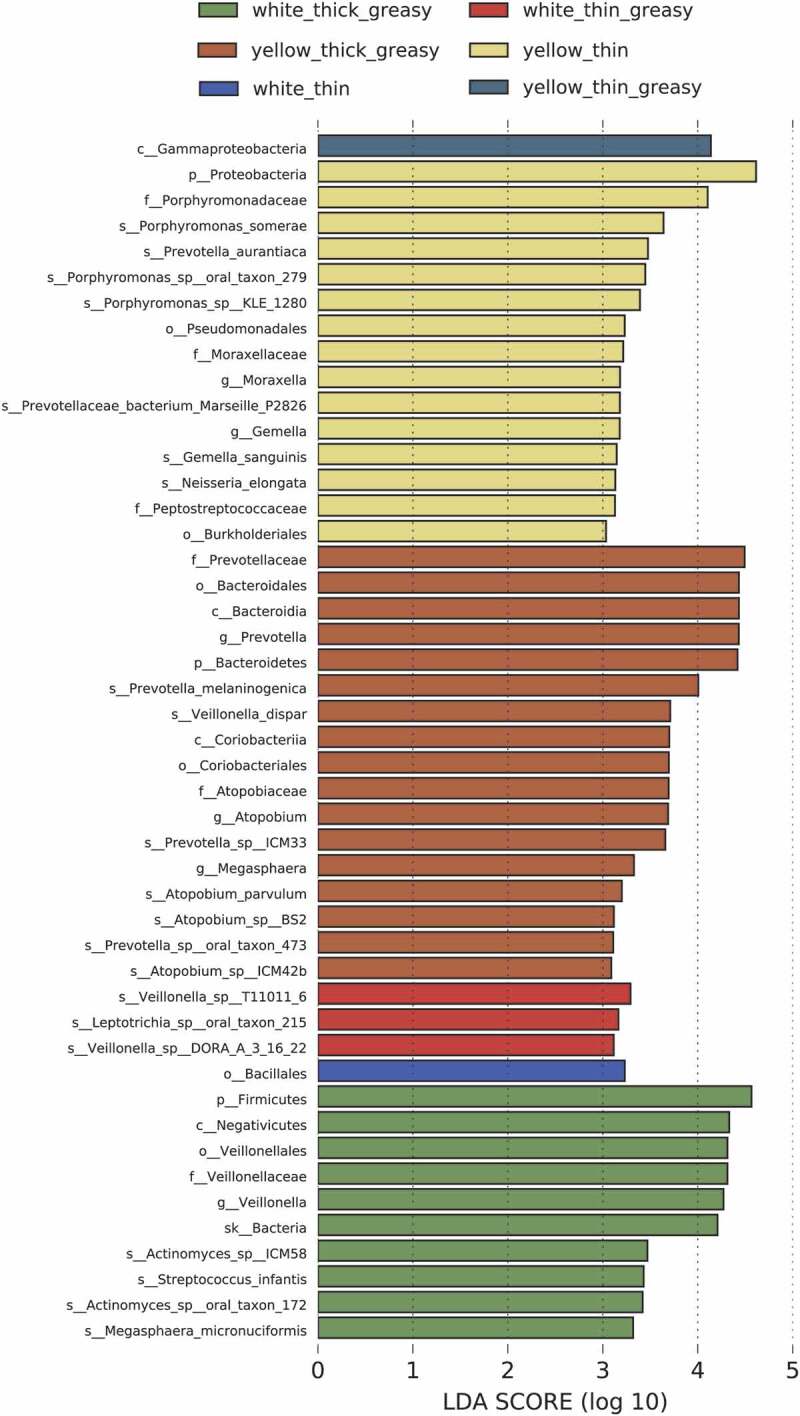
Top differentially enriched taxa in different tongue coating types. Enriched taxa in the designated tongue coating type with LDA scores higher than 3 from LEfSe analysis are presented

Since we did not find a significant difference in microbial beta diversity with respect to different tongue coatings, as revealed by PCA (Supplementary Figure 1), we applied LDA by comparison between each group and the other groups to further identify the specific marker microbes of a specific group (Supplementary Figure 2). The analysis results showed very few bacteria with LDA scores higher than 3 in the W-thin tongue coating, except for the *Bacillales* at order level. For W-thin greasy, three functionally unknown species were found to be abundant (Figure 3). For W-thick greasy, the abundance of *Megasphaera micronuciformis* and members of the *Veillonellaceae* family, as well as the *Firmicutes* phylum, plus *Streptococcus infantis* under the *Firmicutes* phylum were all found to be enriched. Moreover, two bacteria related to oral dental caries, *Actinomyces* sp. ICM58 and *Actinomyces* sp. oral taxon 172 belonging to *Actinobacteria*, were enriched in the W-thick greasy group [18].

Additionally, we found that the phylum *Proteobacteria* was enriched in the Y-thin tongue coating group. Within *Proteobacteria*, the *Moraxella* genus and *Porphyromonas*somerae, as well as *Porphyromonas* sp. KLE 1280, *Porphyromonas* sp. oral taxon 278, and *Porphyromonas* oral taxon 279, all belonging to the family *Porphyromonadaceae*, were highly enriched. Similarly, within the *Bacteroidetes* phylum, *Prevotella* genus, the human oral cavity resident species *Prevotella aurantiaca* were more abundant in the Y-thin group, while in the phylum *Firmicutes*, the species *Gemella sanguinis*, together with upper class *Gemella* at the genus level and members of the family *Peptostreptococcaceae*, were enriched. For the Y-thick greasy tongue coating group, *Bacteroidetes* was the most abundant phylum, and at the species level, *Prevotella melaninogenica* was highly enriched. In *Firmicutes*, the species *Veillonella dispar*, which mostly resides on the tongue dorsum, and *Atopobium parvulum* and its closely related species *Atopobium* sp. BS2 and *Atopobium* sp. ICM42b, which belong to the *Actinobacteria* phylum, were highly abundant in the Y-thick greasy group (Figure 3).

### Disease-related characteristic bacteria in specific tongue coating types

We then carried out an in-depth literature search and confirmed that among the enriched bacteria in specific tongue coating types, many are indicated in certain diseases (these are summarized in Table 2). In the W-thick greasy type, the associated microbial biomarkers *Vellonella, M. micronuciformis* and *Streptococcus infantis* are associated with gastritis. Specifically, the genus *Veillonella* is causative for gastritis of a specific subtype [9]. *M. micronuciformis* is enriched in the tongue coating of gastritis cancer patients [12], while *S. infantis* indicates the precancerous cascade [19]. Moreover, the white coating tongue appearance has been demonstrated to be enriched in gastritis patients in a cohort of 4,742 individuals by logistic regression analysis, making this appearance indicative of a higher risk of gastritis [6].

**Table 2.** Disease related characteristic bacteria in specific tongue coating types

Tongue coating	Marker family/genus/species	Associated diseases	Reference
White thick greasy	*Veillonella*	gastritis	9
	*Megasphaera micronuciformis*	gastritis cancer	12
	*Streptococcus infantis*	gastritis; pancreatic cancer	19
Yellow thin	*Moraxell*a	opportunistic infection	20
	*Neisseria elongata*	pancreatic cancer, endocarditis	13, 21–23
	*Gemella sanguinis*	endocarditis	24
	*Peptotreptococcaceae*	colorectal cancer	25
	*Porphyromonas somerae*	soft tissue infection	26
Yellow thick greasy	*Prevotella melaninogenica*	esophageal and oral squamous cell carcinoma, periodontitis, Alzheimer’s disease mortality, hyperglycemia, infected salivary glands	27–32
	*Veillonella dispar*	esophageal squamous cell carcinoma	27
	*Atopobium parvulum*	sepsis	33

Within the biomarker of the Y-thin tongue coating type, the genus *Moraxella* and species *Neisseria elongate* are common bacterial microbiota of the oropharynx. *Moraxella* sometimes gives rise to opportunistic infections [20], and numerous reports indicate endocarditis [21,22], osteomyelitis [23] and pancreatic cancer [13] induced by *Neisseria elongata*, typically arising after dental procedures or dental abscesses. In *Firmicutes*, the species *G. sanguinis* was again an opportunistic pathogen leading to endocarditis [24], and the family *Peptostreptococcaceae* was found to be overrepresented in the guts of colorectal cancer patients [25]. Finally, the enriched *Bacteroidetes* species *P. somerae* is related to soft tissue infection [26].

The esophageal squamous cell carcinoma-inducing oral bacteria *P. melaninogenica* and *V. dispar* [27] were coenriched in the Y-thick greasy group. Multifunctional *P. melaninogenica* in saliva is also associated with oral squamous cell carcinoma [28] and is causative of periodontitis [29], Alzheimer’s disease mortality [30], hyperglycemia [31], [and MHCII, CD80 and IFN-γ upregulation in salivary gland epithelial cells in Sjögren’s syndrome [32]. The species *A. parvulum* may be associated with sepsis [33]. Moreover, the yellow tongue coating was revealed to be a tongue coating appearance that can predict esophagitis [6].

### Bacterial correlational network within the tongue coating microbiome

To identify the potential correlations and thus the interactions of marker bacteria within each group, correlation analysis was carried out (Table 3). We identified interesting correlational networks that might indicate important bacterial cofunctions within microbiomes of different tongue coating types. Our analysis showed that the highly enriched gastritis-related *S. infantis* in the W-thick greasy group was positively associated with *Streptococcus peroris* with undetermined pathogenic potency. These two bacteria have been found to coexist on the tooth surface and in the pharynx of humans [34]. Within the Y-thin tongue coating bacteria, the representative Y-thin bacterium *N. elongata* was positively correlated with *C. sputigena*, a pathogenic species associated with horioamnionitis, abortion, periodontitis, pleuropneumonitis [35,36,37,38] and abscess [39,40], and with the oral cavity resident Lautropia mirabilis [41–44]. In the Y-thick greasy group, the abundant species *P. melaninogenica, V. dispar* and *Atopobium parvulum* were significantly positively correlated with each other, demonstrating the coexistence and potential interactions of these bacteria. *P. melaninogenic*a and *V. dispar* were both positively correlated with *Prevotella fusca*, while *P. melaninogenica* and *A. parvulum* were both positively associated with *Prevotella scopos. P. fusca* and *P. scopos* coexist in human oral cavity and *P. fusca* is implicated in periodontitis [45,46] and can serve as additional marker bacteria for the Y-thick greasy tongue type.

**Table 3.** Co-occurrence of enriched bacteria in different tongue coating types

Tongue coating type	Marker species	Co-occurring bacteria	R	p-value
White thick	*Streptococcus infantis*	*Streptococcus peroris*	0.99300699	1.30E-10
Yellow thin	*Neisseria elongata*	*Capnocytophaga sputigena*	0.88571429	0.01884548
	*Neisseria elongata*	*Lautropia mirabilis*	0.88571429	0.01884548
Yellow thick greasy	*Prevotella melaninogenica*	*Veillonella dispar*	0.70909091	0.02166592
	*Prevotella melaninogenica*	*Atopobium parvulum*	0.75757576	0.01114345
	*Prevotella melaninogenica*	*Prevotella scopos*	0.89090909	0.00054214
	*Prevotella melaninogenica*	*Prevotella fusca*	0.81818182	0.00381492
	*Veillonella dispar*	*Atopobium parvulum*	0.63636364	0.04791173
	*Veillonella dispar*	*Prevotella fusca*	0.6969697	0.02509668
	*Atopobium parvulum*	*Prevotella scopos*	0.87878788	0.00081386

To further investigate the underlying reason why different tongue coatings were characterized by different bacteria, the unstimulated salivary flow rate, which impacts the oral microbiota abundance and variation [47,48], was examined in additional individuals with the different tongue coating types (Supplementary Table 1). The results showed that individuals with thick tongue coatings tended to have slower salivary flow rates than those with thin tongue coatings (Supplementary Figure 3), and more than half of the participants with thick tongue coatings demonstrated hyposalivation (< 0.1 g/min). Specifically, the flow rate of the Y-thick tongue coating group decreased significantly compared with that of the white thin and yellow thin tongue coating types (Supplementary Figure 3). This indicates a potential role of the salivary flow rate in influencing the microbiome in thick tongue coating groups, especially in the Y-thick tongue coating group.

### Type 2 diabetes bacterial markers in the tongue coating microbiome are distinct from those in the healthy population

**Figure 4.**
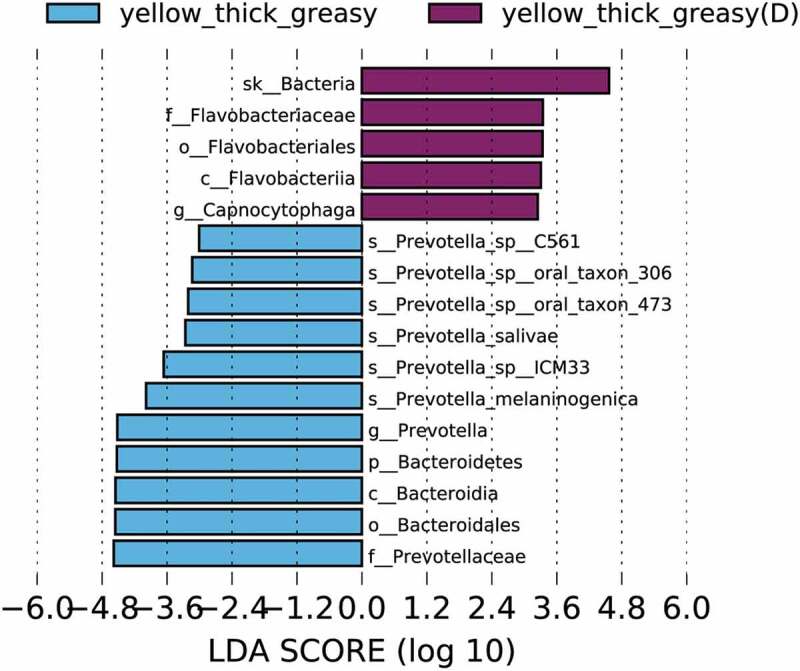
Top differentially enriched taxa in the Y-thick greasy tongue coating of healthy and diabetic populations. Enriched taxa in the designated tongue coating type with LDA scores higher than 3 in LEfSe analysis are presented

Because most existing studies focusing on tongue coating marker bacteria were carried out in the context of specific diseases, it is difficult to distinguish specific tongue coating microbiomes from the interferences of diseases. To investigate the potential differences in the same tongue coating between healthy individuals and individuals with disease, we carried out a primary comparative study of the tongue coating microbiome in the same Y-thick greasy group in the healthy group and in an additional diabetic group. To our surprise, the microbiota in the two groups showed clear distinctions (Supplementary Figure 4, p = 0.098). In the Y-thick greasy group of diabetic patients, the *Capnocytophaga* genus was highly enriched (Figure 4). This potentially pathogenic genus is reported to be related to periodontitis and type 1 diabetes mellitus [49], and it was recently found in a diabetic patient with peritonitis [50]. The species *P. melaninogenica* was enriched in the healthy population. Such differences in microbe composition were still present between sex-matched groups, which included four males from the healthy Y-thick greasy group and four males from the diabetic Y-thick greasy group (Supplementary Figure 5A). Specifically, *Capnocytophaga* again was indicative of diabetic patients, together with *N. elongata* and *Neisseria mucosa*, which may be specific to male diabetic patients (Supplementary Figure 5B). Therefore, many of the previous disease-based studies might be misleading and cannot offer reliable baseline information on the tongue coating microbiome, thereby potentially causing confusion in many related interpretations.

## Discussion

In our current study, we profiled the tongue coating microbiome in healthy populations and demonstrated their differences with respect to physical appearances, a judging criterion used in TCM throughout history. The consistency of marker microbes in our study compared with previous reports demonstrates the stable residence of these microbes in the tongue coating. The most common phyla identified in our study, *Firmicutes, Bacteroidetes, Proteobacteria, Actinobacteria* and *Fusobacteria* (Figure 2), steadily reappeared in other reports [11,15,19]. In addition, we did not find major differences in composition among respective tongue coating types by overall comparison (Supplementary Figure 1), which is in agreement with previous reports that low beta-diversity differences (between samples) were found in oral samples of healthy populations [15,51,52].

Despite finding significant bacterial markers only in three tongue coating groups, these marker species are distinct from those in previous studies. Among the four potential biomarkers of the W-thick coating in gastric cancer patients [12], only *M. micronuciformis* reappears in our study. As *M. micronuciformis* and *Veillonella* were repeatedly found to reside in W-thick coatings in gastric inflammation patients [9], gastritis cancer patients [12] and the healthy population (in our study), they may serve as significant distinguishable microbes for W-thick tongue coatings. The W-thick coating tongue appearance indeed can predict stomach dysfunction [5,6], and *M. micronuciformis* and *Veillonella* may thus serve as pre-gastritis markers in the healthy population. Moreover, whether and how these species contribute to the formation of W-thick tongue coatings is worthy of further investigation. [*Proteobacteria* was higher in abundance in the Y-thin group than in the Y-thick group in gastric cancer patients [12] and was enriched in the Y-tongue coating of chronic hepatitis B patients [11]. In our study, the Y-thin group similarly had the highest level of *Proteobacteria* (Figure 3). Within this phylum, we further pinpointed the enrichment of the genus *Moraxella* and the species *N. elongata* in Y-thin coatings, which was not identified previously. *Bacteroidetes* was upregulated in the Y-thick group in our study but decreased in chronic hepatitis B patients, indicating that HBV may drive the declination of *Bacteroidetes* [11]. Moreover, for the Y-thick greasy tongue coating, the enrichment of cancer and sepsis associated *P. melaninogenica, V. dispar* and *A. parvulum* suggested the potential occurrence of these serious diseases, which requires close follow-up. Coincidentally, the yellow tongue coating population is at a high risk for esophageal cancer [6]. Finally, the significant enrichment of the diabetic marker bacteria *Capnocytophaga* in the Y-thick greasy group of diabetic patients emphasizes the importance of our analysis and the need to distinguish disease-specific and tongue coating-specific bacteria in future studies (Figure 4). Collectively, the identified bacterial markers together with the tongue coating appearance in the healthy population may indicate potential dysfunction of specific organs and the occurrence of specific diseases, which requires further health monitoring follow-up study of the healthy population.

The reasons behind different tongue coating types enriched by distinctive marker microbes may partly be attributed to different salivary flow rates [53]. It has been reported that more frequent self-reported symptoms of low salivary flow rate was associated with higher abundance of tongue coating *P. melaninogenica* [54]. Consistently, we demonstrated that the Y-thick greasy group had significantly less salivary secretions (Supplementary Figure 3) and that *P. melaninogenica* was distinguishable as the characteristic marker microbe (Table 2). However, although the salivary flow rate seems to decline in the W-thick greasy group (Supplementary Figure 3), the characterized marker microbe, *M. micronuciformis* (Table 2), is not reported to be associated with impaired salivary secretion [55]. Therefore, how the salivary flow rate affects each group and, in particular, different marker species requires further studies and interpretations.

More investigations and in-depth studies need to be performed to determine the mechanism leading to different tongue coating appearances. It is unknown whether the characteristic bacteria and their associated partners shown by our comparative analysis would be sufficient to produce factors such as pigment or metabolic molecules to form a specific color or thickness on the tongue. For example, *Capnocytophaga* or *P. melaninogenica* alone may be dispensable for the Y-thick greasy appearance (Figure 4), as the Y-thick greasy appearance could occur with either of these strains. On the other hand, nutrient acquisition from host epithelial material and from the oral cavity through saliva, as well as interaction with neighboring microbes, are essential for the patch-like growth of bacteria [3]. Additionally, apoptotic epithelial cells may contribute to different tongue coatings [56].

Taken together, the results of this study revealed the characteristic microbiome of distinctive tongue coatings in a healthy population, which sheds light on the tongue coating formation mechanism. This study also provides a valuable reference for investigating disease-specific tongue coating bacterial markers for future studies.

## Supplementary Material

Supplemental Material
