# Supplementary material for: Microbial characteristics across different tongue coating types in a healthy population
Source: J Oral Microbiol. 2021 Jul 26;13(1):1946316. doi: 10.1080/20002297.2021.1946316 (PMC8317956; doi:10.1080/20002297.2021.1946316)
Supplement: Supplemental Material [file ZJOM_A_1946316_SM3021.zip › Supplementary/Supplementary Material1.docx]

**Supplementary Material**

**Microbial characteristics across different tongue coating types in a healthy population**


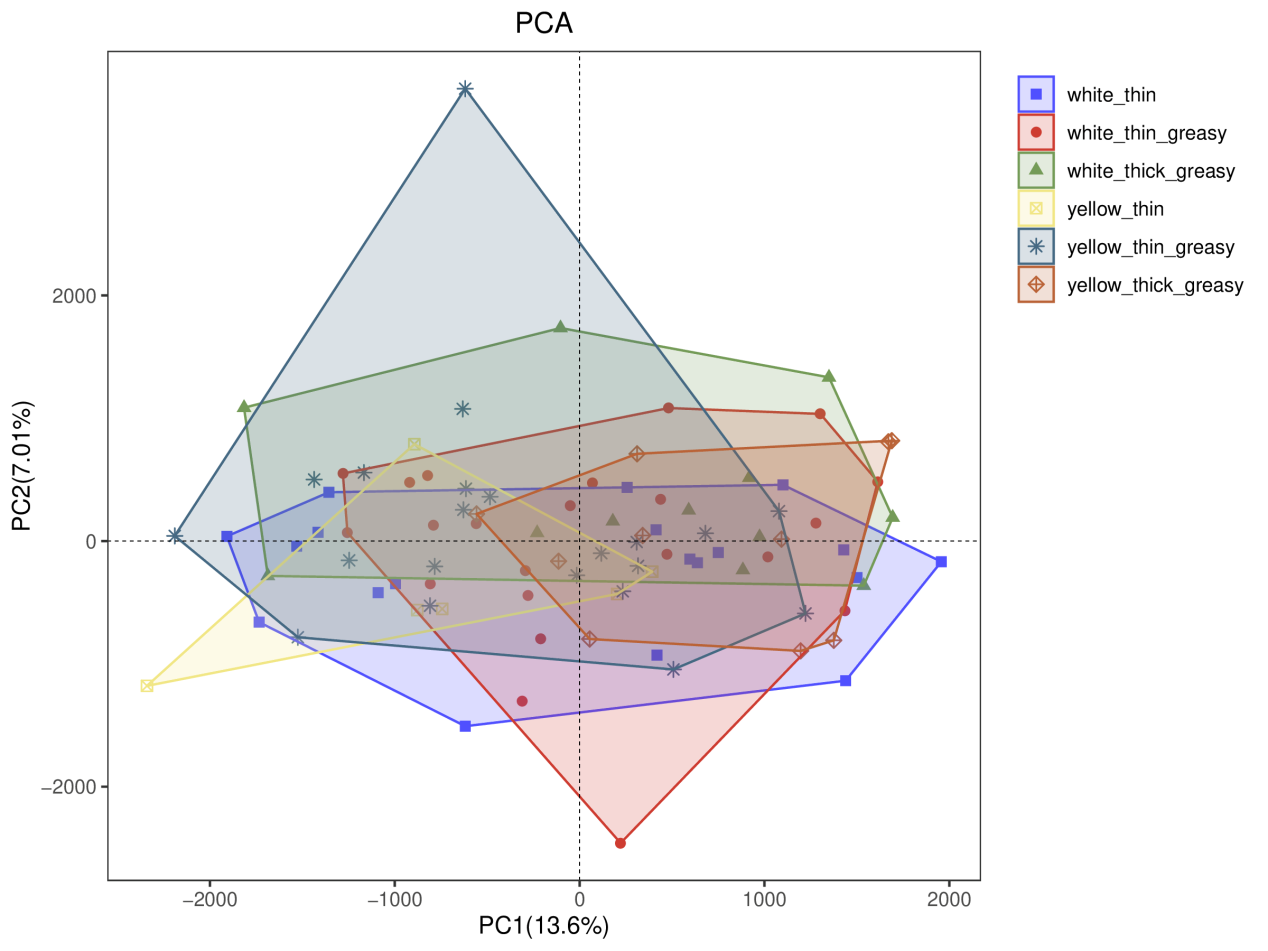
**Supplementary Fig. 1.** PCA plot of bacteria in different tongue coating of all 94 samples.


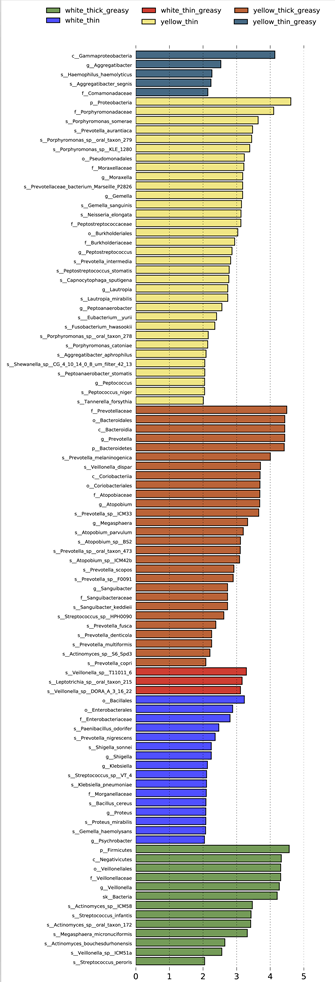


**Supplementary Figure 2.** Top differentially enriched taxa in different tongue coating types. Enriched taxa in designated tongue coating type with LDA scores higher than 2 were presented.

| **Supplementary Table 1.** Information of participants taking part in salivary flow rate experiments | | | | |
| --- | --- | --- | --- | --- |
|  | white thin | white thick greasy | yellow thin | yellow thick greasy |
| Number of cases | 6 | 4 | 5 | 5 |
| Age | 40.83±2.09 | 50.25±5.85 | 43.60±6.89 | 52.60±3.72 |
| Males(%) | 3(50%) | 3(75%) | 3(60%) | 3(60%) |

Data are presented as mean ± SEM.

**Supplementary Figure 3.** Unstimulated salivary flow rate in different tongue coating types. Data are mean ± SEM, Student t-test. *p <0.05


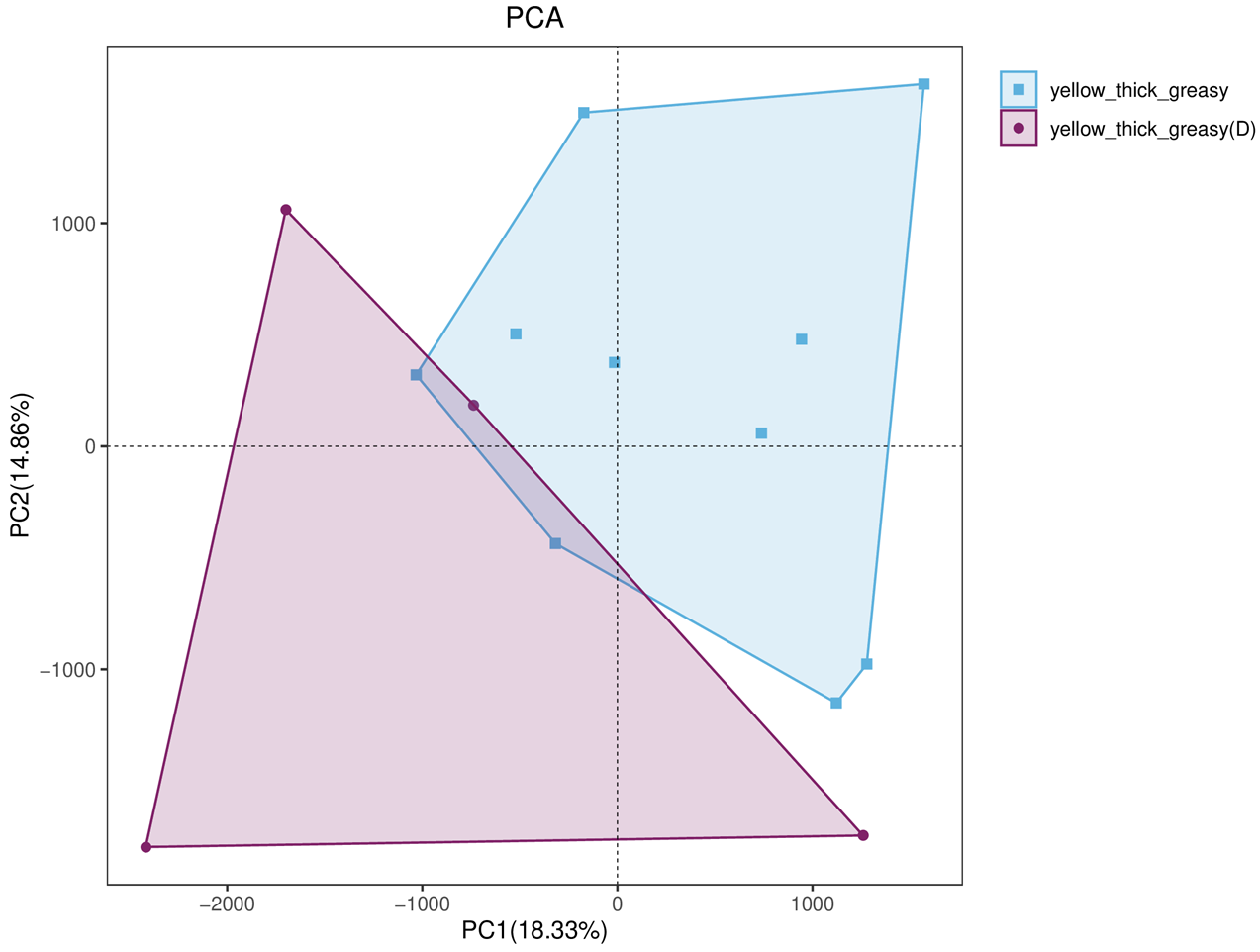


**Supplementary Figure 4,** PCA plot of bacteria in Y-thick greasy tongue coating of healthy and diabetic population. Significance of separation of PCA is calculated by Anosim, p = 0.098.


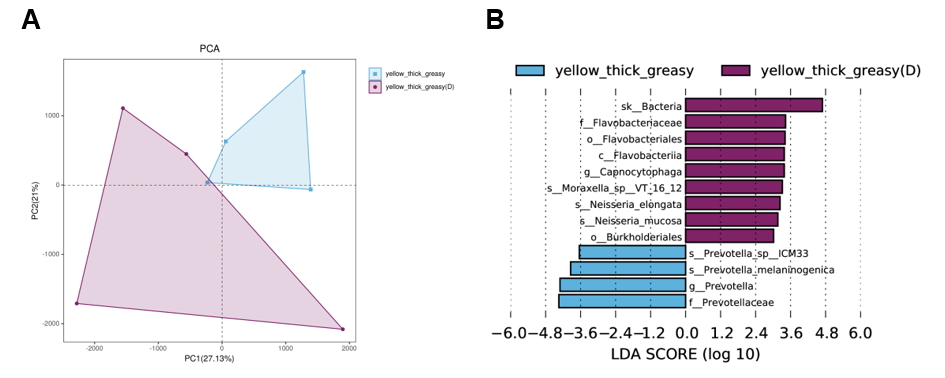


**Supplementary Figure 5.** PCA plot and top differently enriched taxa in yellow thick greasy tongue coating in males of health and of a diabetic group. Significance of separation of PCA is calculated by Anosim, p = 0.211.
